# Supplementary material for: An automated Bayesian pipeline for rapid analysis of single-molecule binding data
Source: Nat Commun. 2019 Jan 17;10:272. doi: 10.1038/s41467-018-08045-5 (PMC6336789; doi:10.1038/s41467-018-08045-5)
Supplement: Supplementary file 1 — Supplementary Information [file 41467_2018_8045_MOESM1_ESM.pdf]

Supplementary Information

An Automated Bayesian Pipeline for Rapid Analysis of Single-Molecule Binding Data

Smith et al.

Supplementary Figure 1 | Comparison of the Pipeline to Imscroll.

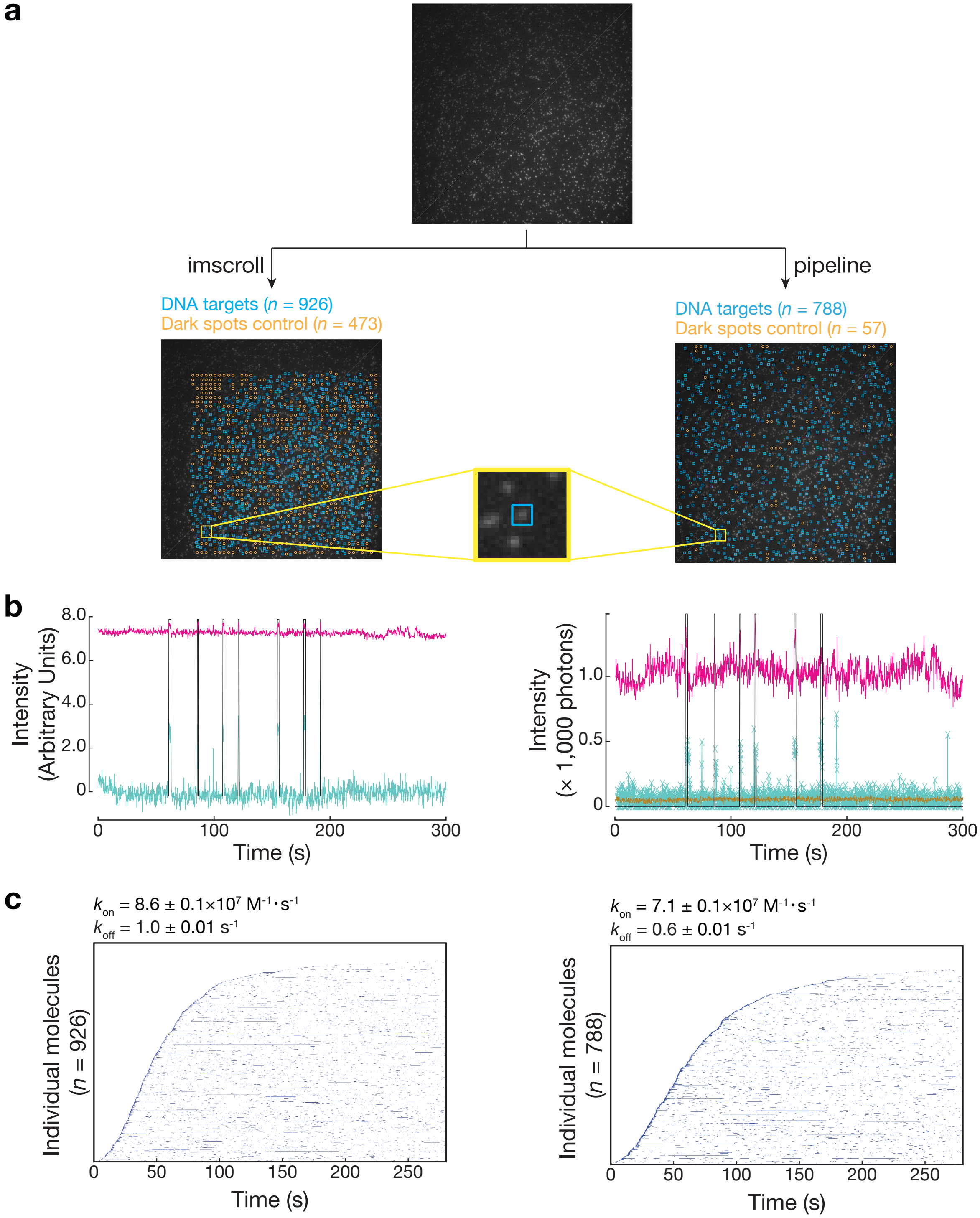

The same data set of TtAgo:guide complex binding a seed-matched target was analyzed by imscroll **(a)** and by the pipeline **(b)**. Image representing the selection (blue squares around molecules) of the DNA targets used to analyze DNA-guided TtAgo binding. Dark locations, i.e., regions that contained no target molecules (orange circles) served as a control for non-specific binding of TtAgo:guide complex to the surface of the cover glass. Representative fluorescence intensity time traces obtained by imscroll **(a)** or the pipeline **(b)** for DNA-guided TtAgo (turquoise) binding the same DNA target molecule (magenta). The black line denotes detected binding events. Light brown indicates background levels of green fluorescence calculated by the pipeline. Imscroll does not provide this information. **(c)** Rastergrams summarize traces of individual target molecules, each in a single row and sorted according to their arrival time. Values of k_{on} and k_{off} were derived from several hundred individual DNA target molecules; standard error from bootstrapping is reported.

Supplementary Figure 2 | DNA-Guided TtAgo Binding to and Departing from DNA Targets at Different Temperatures.

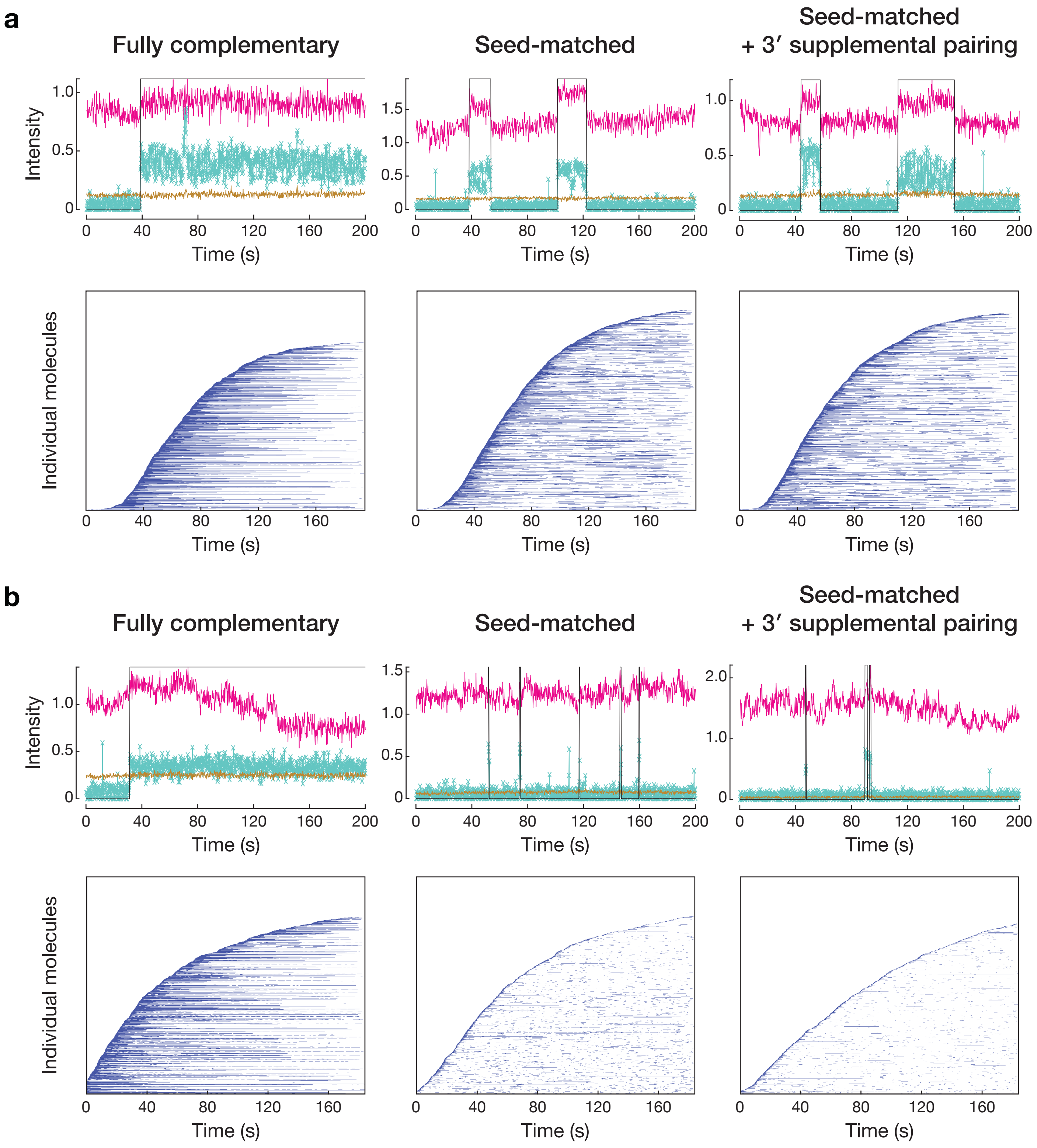

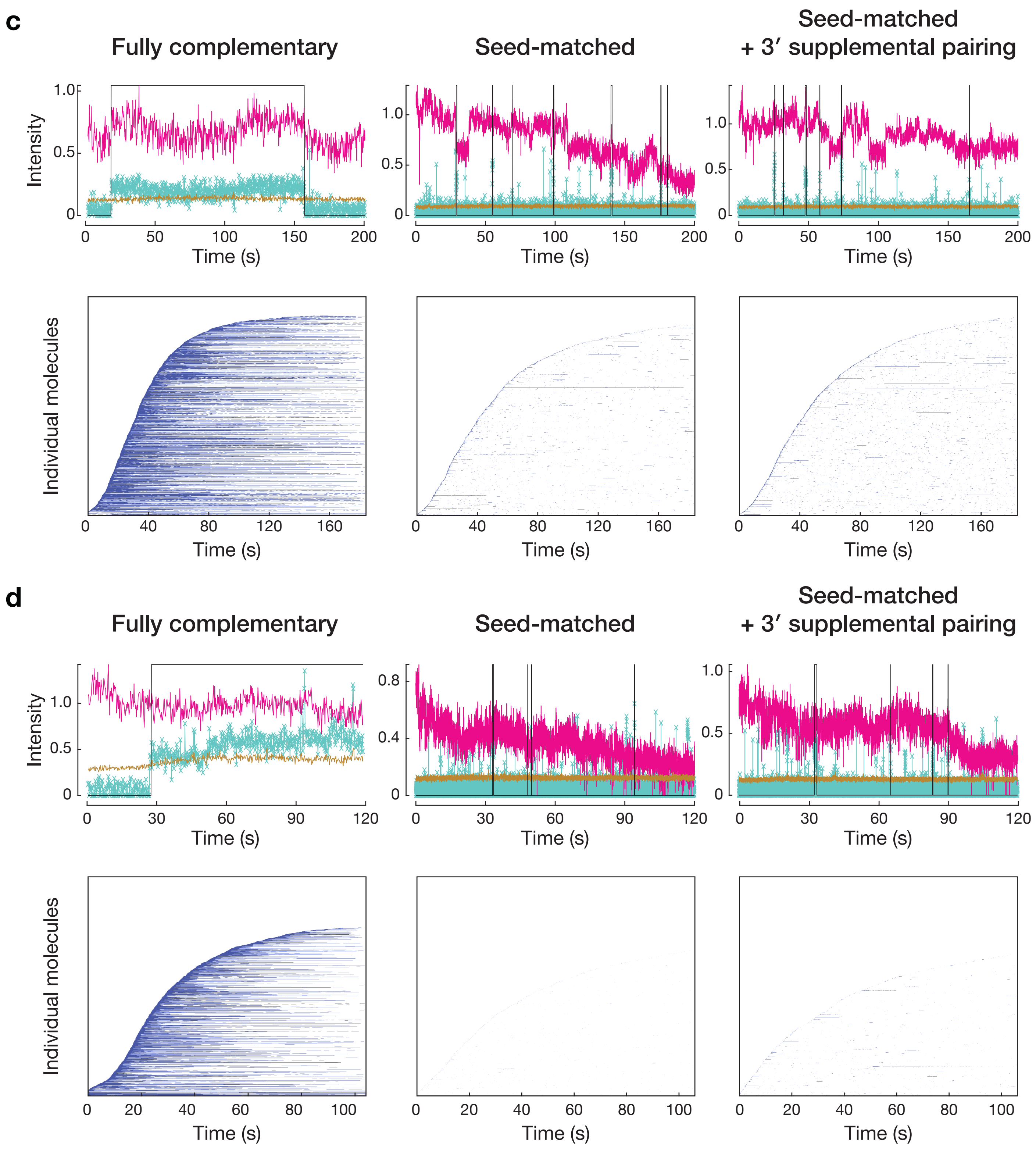

Representative fluorescence intensity time traces of DNA-guided TtAgo (turquoise) binding DNA target (magenta) with different extents of complementarity. Light brown indicates background levels of green fluorescence, whereas the black line denotes binding events detected by the pipeline after event filtering (minimal duration and gap closing; see **User Manual** – Co-localization analysis). Fluorescence intensity is expressed in thousands of photons. Rastergrams summarize traces of individual target molecules, each in a single row and sorted according to their arrival time, for different guide:target pairings. Experiments were performed at 23°C **(a)**, 37°C **(b)**, 45°C **(c)** and 55°C **(d)**.

Supplementary Figure 3 | Custom-Fabricated Sample Heater for High-Temperature Experiments.

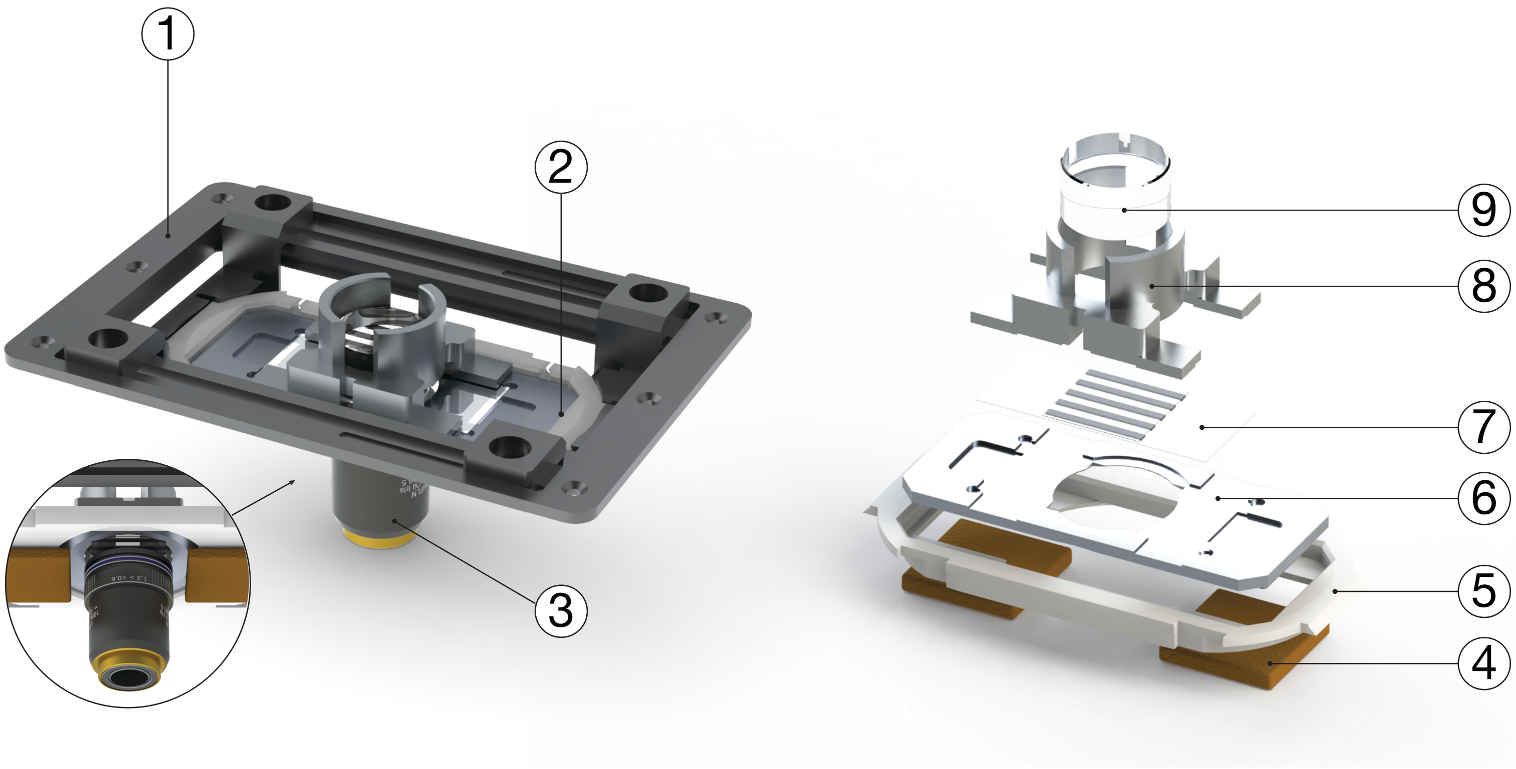

Clamped inside the microscope sample holder **(1)**, the heating assembly **(2)** stably positions the temperature-controlled sample over the objective **(3).** PID (proportional–integral–derivative) controlled surface heating pads **(4)** heat the aluminum holder **(6)** for the sample slide **(7)**, as well as the thermally conducting aluminum holder **(8)** for the optical window **(9).** The mounting adapter **(5)** thermally insulates the heating assembly from the microscope body while a heating collar for the objective (not shown) minimizes the thermal gradient over the sample. Drawings and CAD-models of the custom parts and the stage-heater assembly are available at https://www.thingiverse.com/thing:2791422.

Supplementary Figure 4 | Simulation of Single-Molecule Switching Kinetics and Prediction of the Number of States by VBEM-MGHMM.

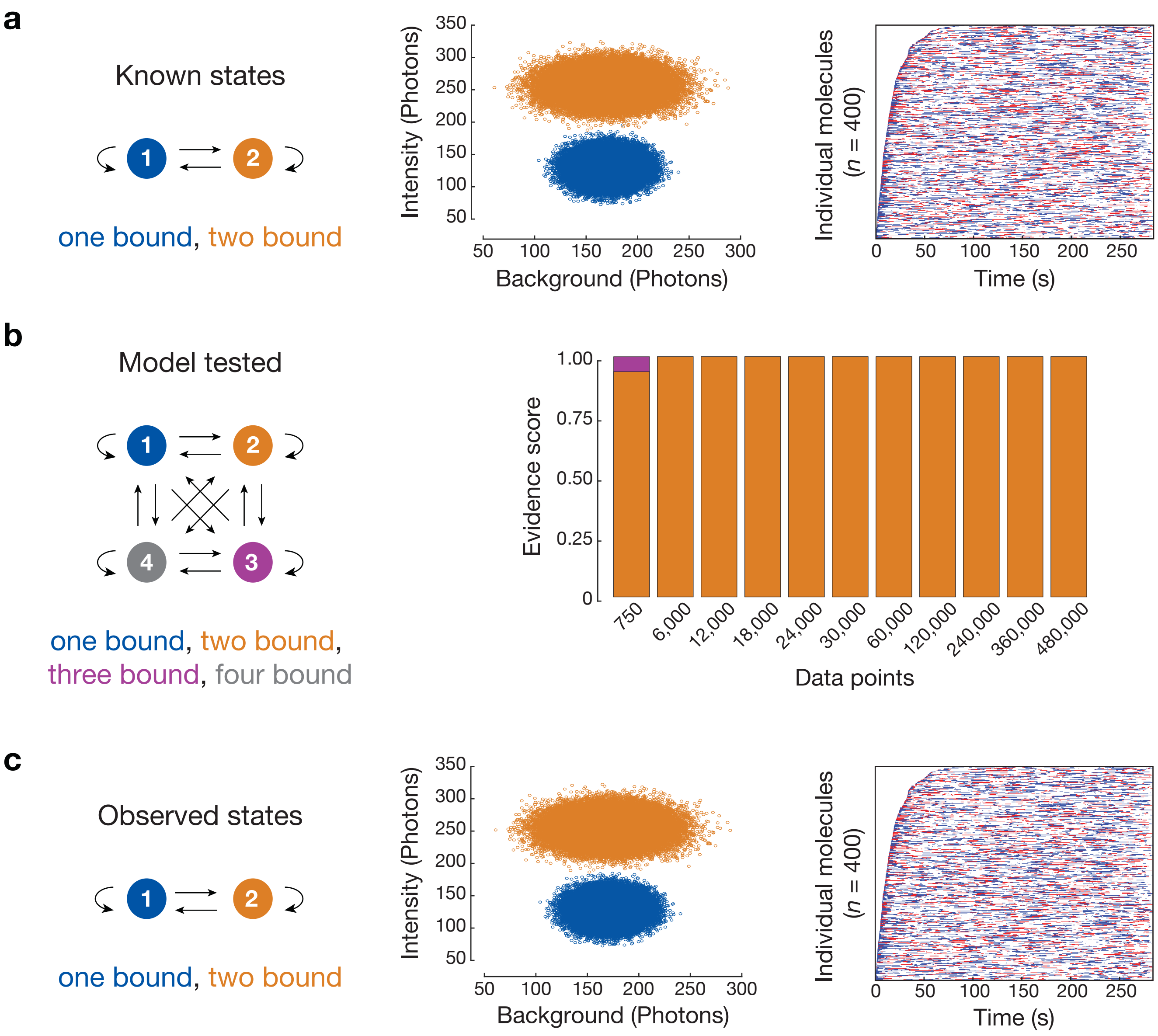

(a) Simulated dataset consists of 400 target molecules, which can be bound by one or two molecules of the mobile component during 1,500 frames (representing 300 s). Fluorescent intensities of states 'one bound' and 'two bound' follow Gaussian non-overlapping distributions. Rastergram summarizes traces of individual target molecules, each in a single row and sorted according to their arrival time. **(b)** The simulated dataset containing in total 600,000 data points was bootstrapped and subjected to VBEM-MGHMM analysis setting priors as described in **Supplementary Table 2**. **(c)** Estimated distributions of fluorescent intensities recovered by VBEM-MGHMM analysis are shown for the dataset of 600,000 data points. Estimated binding events are recapitulated in the rastergram.

Supplementary Figure 5 | VBEM-MGHMM Analysis Accurately Recovers the Ground Truth Parameters.

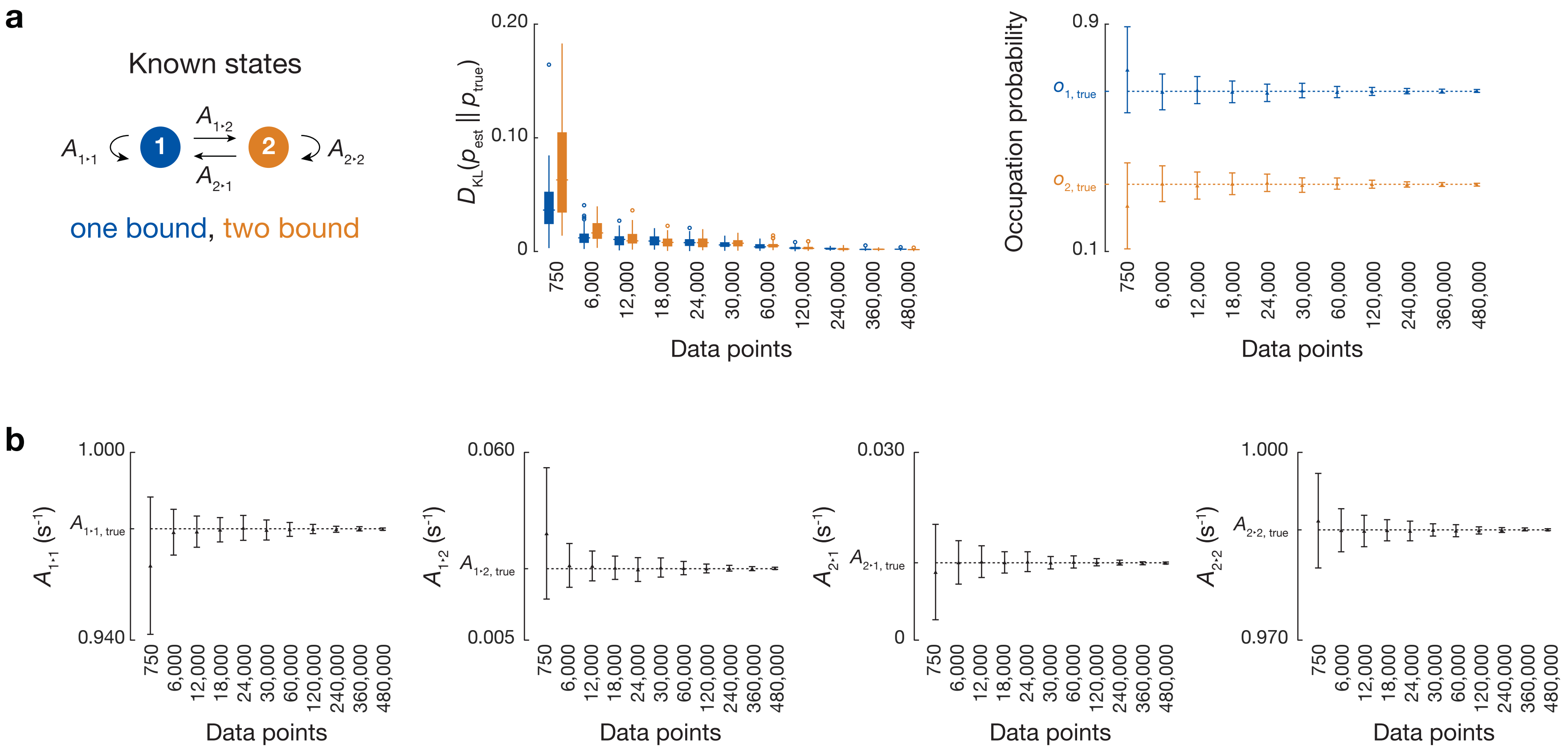

(a) The distributions of fluorescent intensities estimated by VBEM-MGHMM analysis from sub-datasets in **Supplementary Figure 4b** are compared to the true ones from the simulated dataset (**Supplementary Figure 4a**). Occupation probability **(a)** and transition rates **(b)** estimated from sub-datasets asymptotically converge to the true values defined in the simulated dataset. $D_{KL}(p_{est} \| p_{true})$: Kullback–Leibler divergence of estimated probability distribution to the ground truth. o: occupancy, $A_{initial\cdot final}$: transition rate from initial state to final state.

Supplementary Figure 6 | VBEM-MGHMM Algorithm Has a Proneness for Higher Orders.

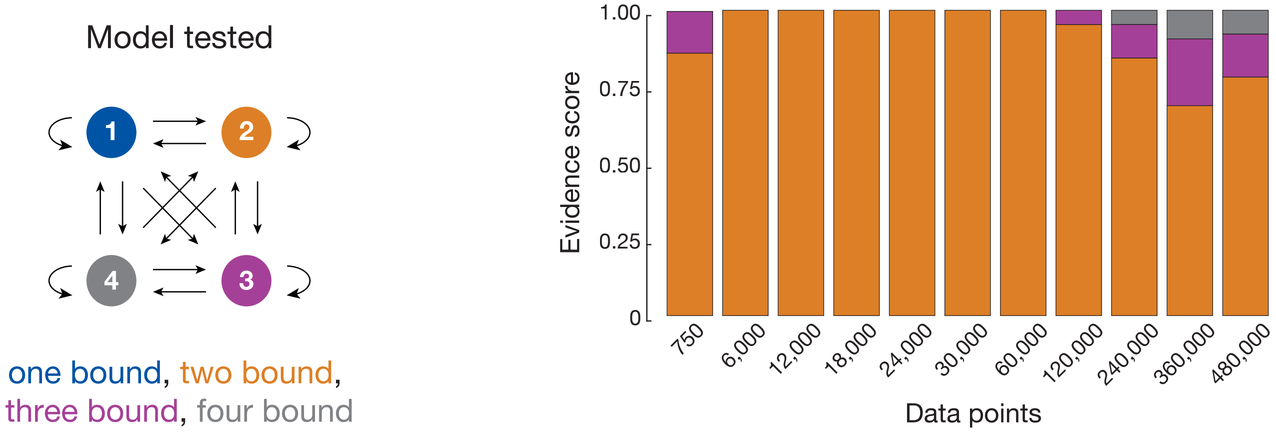

The simulated dataset of 600,000 data points was bootstrapped (**Supplementary Figure 4**) and subjected to VBEM-MGHMM analysis setting aberrant priors on the fluorescent intensity and the background (**Supplementary Table 3**).

Supplementary Figure 7 | DNA-Guided TtAgo Binding to Multiple, Fully Complementary Binding Sites and Prediction of the Number of States by VBEM-MGHMM.

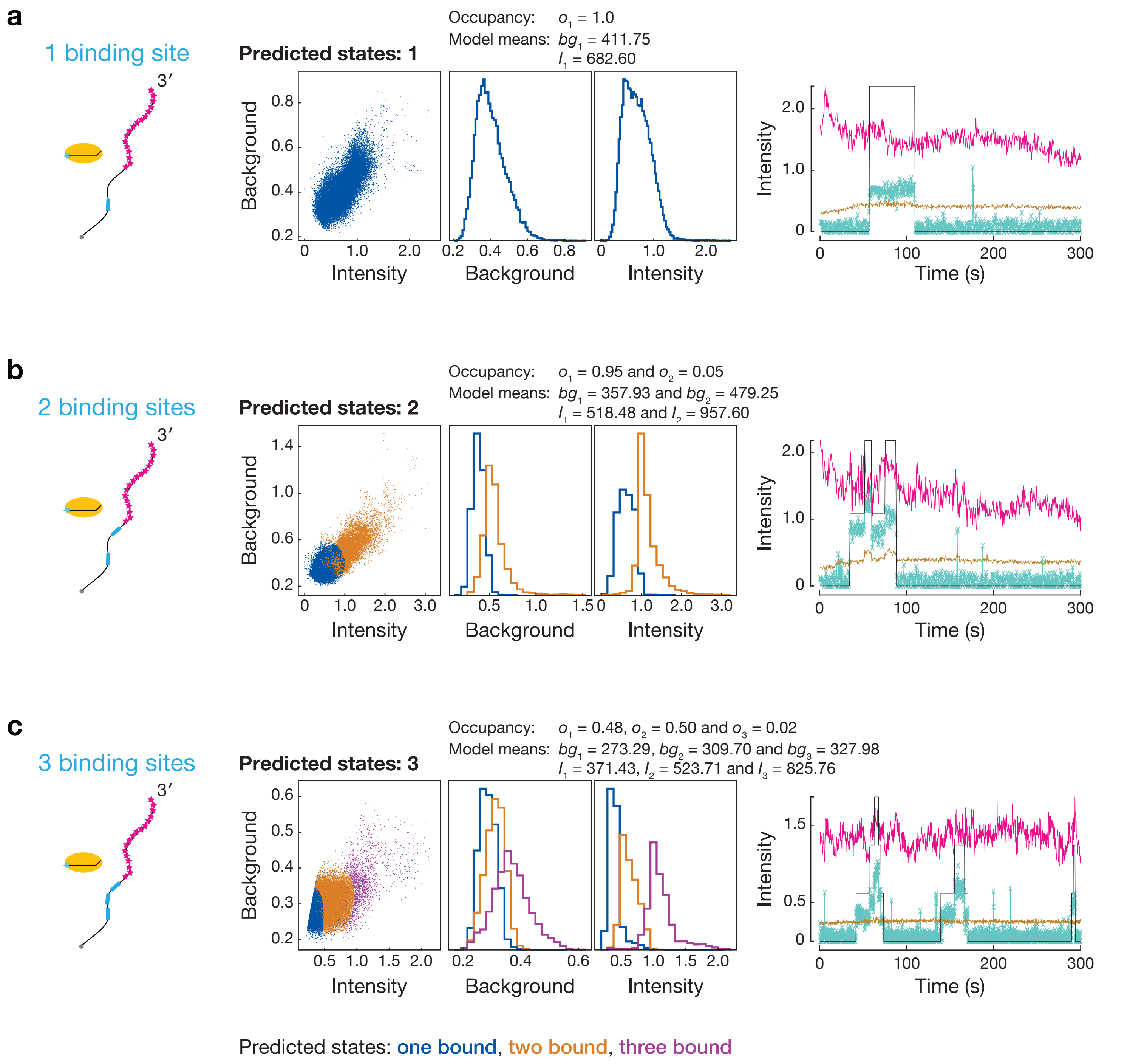

Experimental setup to detect TtAgo:guide interactions with target DNA containing one **(a)**, two **(b)** or three **(c)** binding site(s) complementary to the DNA guide. The number of states was estimated by VBEM-MGHMM. Occupancy (*o*), mean intensity of background (*bg*) and mean intensity of binding event (*I*) are indicated for each state. Representative fluorescence intensity time traces of DNA-guided TtAgo (turquoise) binding DNA target (magenta) are shown. Light brown indicates background levels of green fluorescence, whereas the black line denotes binding events detected by the pipeline after VBEM-MGHMM analysis. Fluorescence intensity is expressed in thousands of photons.

Supplementary Figure 8 | No Simultaneous Binding of TtAgo:Guide Complexes to a Target DNA with Two Seed-matched Binding Sites.

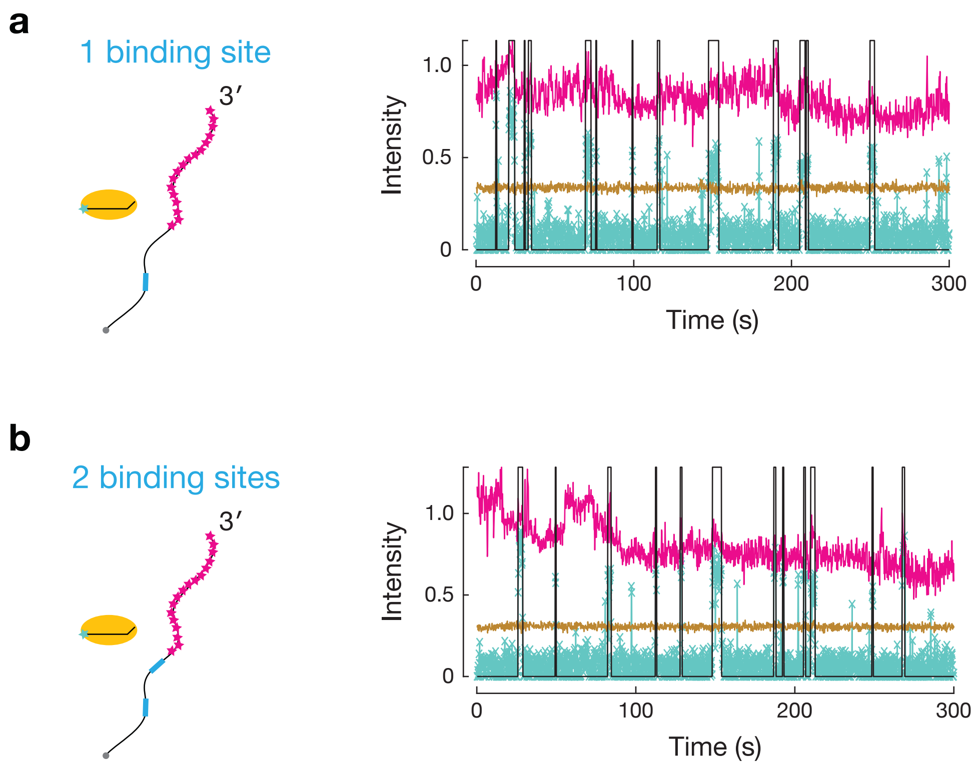

Experimental setup to measure TtAgo:guide complex interactions with target DNA containing one **(a)** or two **(b)** seed-matched binding site(s). Representative fluorescence intensity time traces of DNA-guided TtAgo (turquoise) binding DNA target (magenta) are shown. Light brown indicates background levels of green fluorescence, whereas the black line denotes binding events detected by the pipeline after event filtering (minimal duration and gap closing; see **User Manual** – Co-localization analysis)**.** Fluorescence intensity is expressed in thousands of photons.

Supplementary Figure 9 | TtAgo:Guide Complex Preferentially Binds to a t1G Target DNA.

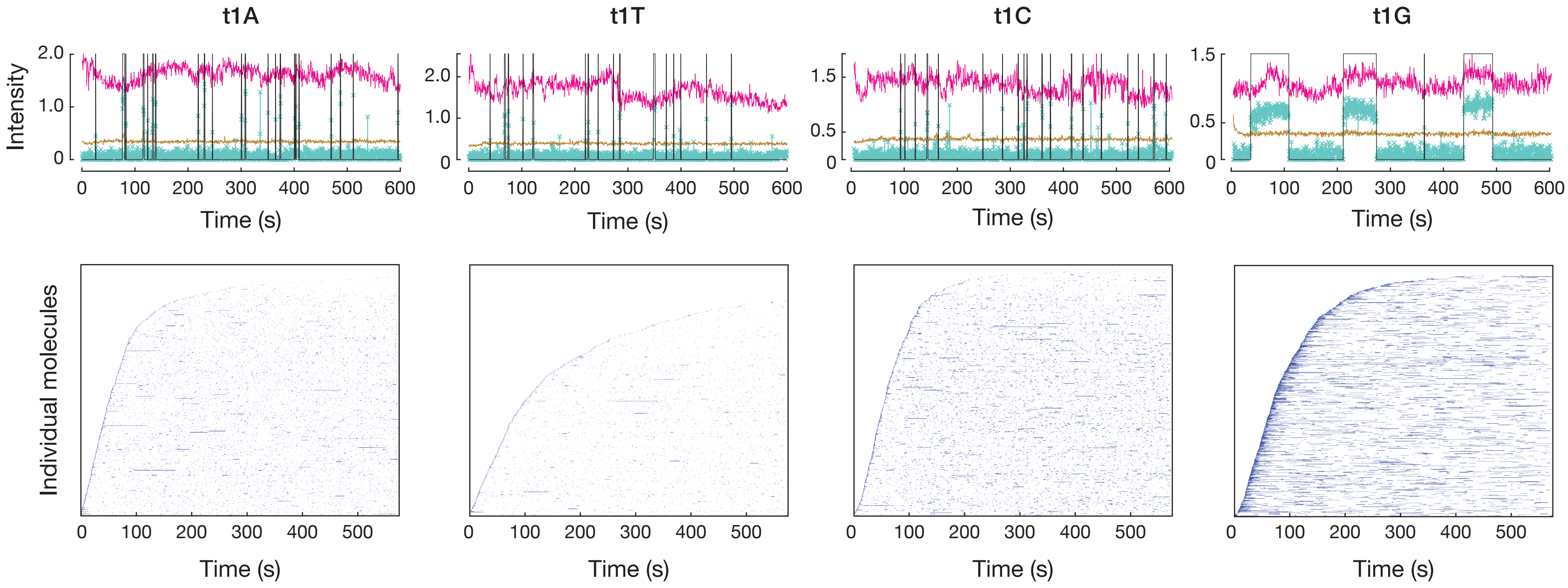

Representative fluorescence intensity time traces of DNA-guided TtAgo (turquoise) binding DNA target (magenta). Light brown indicates background levels of green fluorescence, whereas the black line denotes binding events detected by the pipeline after event filtering (minimal duration and gap closing; see **User Manual** – Co-localization analysis). Fluorescence intensity is expressed in thousands of photons. Shown below are rastergram summaries of traces of individual target molecules, each in a single row and sorted according to their arrival time. Values of k_{on} and k_{off} derived from these data are reported in **Supplementary Table 4.**

Supplementary Figure 10 | DNA-Guided TtAgo Binds Independently to DNA Targets Containing Two Seed-Matched Sites with t1G.

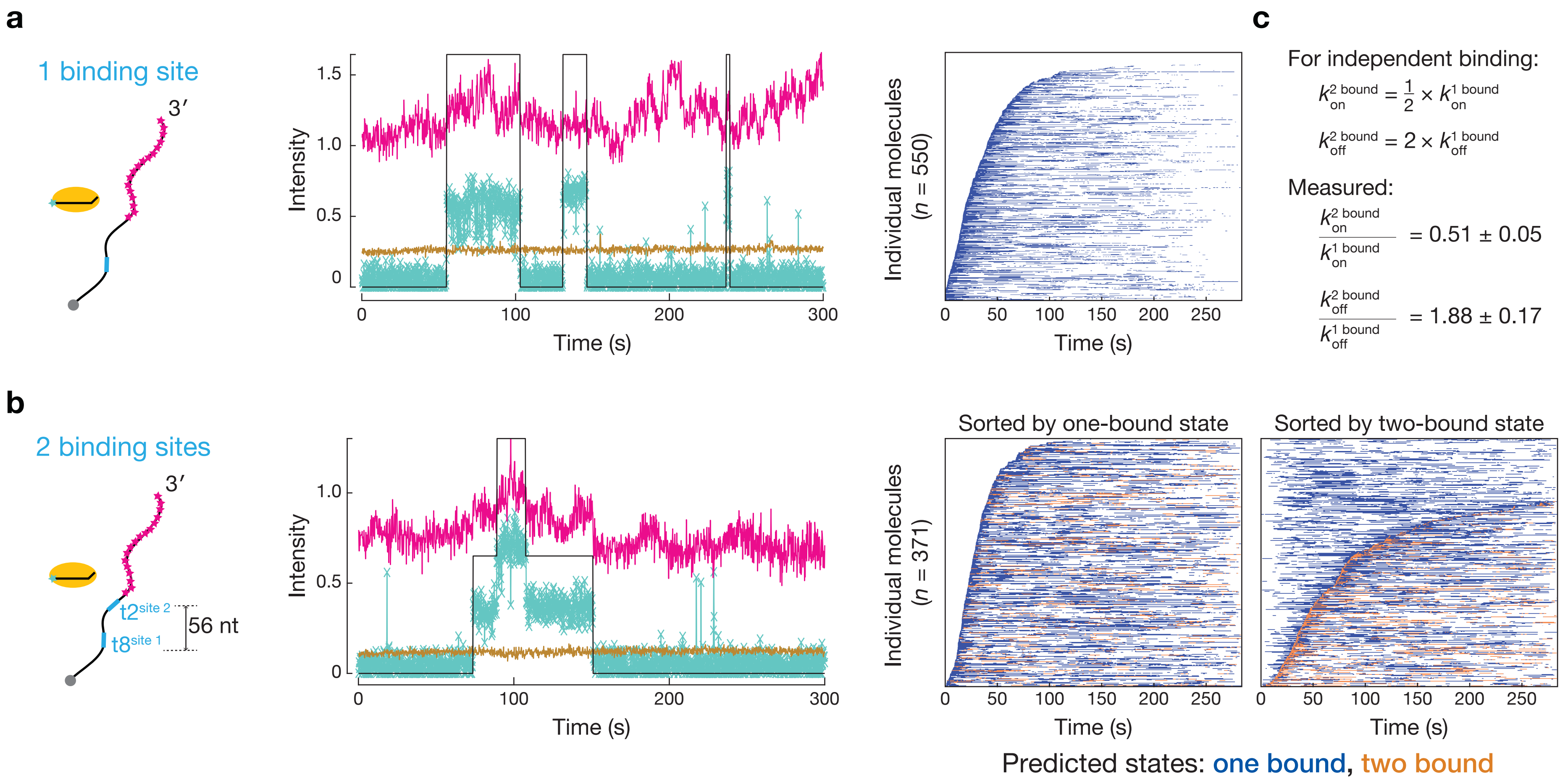

Representative fluorescence intensity time traces of DNA-guided TtAgo (turquoise) binding DNA target (magenta) containing one binding site **(a)** or two binding sites spaced 56 nt apart from t8 to t2 **(b)**. Light brown indicates background levels of green fluorescence, whereas the black line denotes binding events detected by the pipeline after VBEM-MGHMM analysis. Fluorescence intensity is expressed in thousands of photons. Representative rastergrams summarize traces of individual target molecules, each in a single row and sorted according to their arrival time. **(c)** Comparison of k_{on} and k_{off} of DNA-guided TtAgo with targets containing two or one binding site. Values are reported as mean ± standard deviation for three independent replicates.

Supplementary Table 1 | Parameters Used to Simulate Single-Molecule Switching Kinetics.

Parameter	Description	Value
N	Number of states	2
T	Number of traces	400
F	Number of frames per trace	1,500
$A_{1\text{->}1}$	Transition rate state 1 to state 1	0.9756
$A_{1\text{->}2}$	Transition rate state 1 to state 2	0.0244
$A_{2\text{->}1}$	Transition rate state 2 to state 1	0.0124
$A_{2\text{->}2}$	Transition rate state 2 to state 2	0.9876
$\mu_{I,1}$	Mean intensity state 1	171.0312
$\mu_{bg,1}$	Mean background state 1	129.0564
$\mu_{I,2}$	Mean intensity state 2	255.9118
$\mu_{bg,2}$	Mean background state 2	170.6726
$(\Sigma_{I,I})_1$	Variance of the intensity state 1	235.4636
$(\Sigma_{I,bg})_1$	Covariance of the intensity and background state 1	0
$(\Sigma_{bg,I})_1$	Covariance of the intensity and background state 1	0
$(\Sigma_{bg,bg})_1$	Variance of the background state 1	188.1568
$(\Sigma_{I,I})_2$	Variance of the intensity state 2	699.0072
$(\Sigma_{I,bg})_2$	Covariance of the intensity and background state 2	0
$(\Sigma_{bg,I})_2$	Covariance of the intensity and background state 2	0
$(\Sigma_{bg,bg})_2$	Variance of the background state 2	287.8775

Supplementary Table 2 | Priors Used for Order Selection by VBEM-MGHMM Analysis of the Simulated Dataset.

Parameter	Description	Value
N	Maximum order tested	4
$\mu_{I,1}$	Mean intensity state 1	100
$\mu_{I,2}$	Mean intensity state 2	500
$\mu_{I,3}$	Mean intensity state 3	1,000
$\mu_{I,4}$	Mean intensity state 4	1,500
$\mu_{bg,1}$	Mean background state 1	200
$\mu_{bg,2}$	Mean background state 2	200
$\mu_{bg,3}$	Mean background state 3	200
$\mu_{bg,4}$	Mean background state 4	200
κ	Variance of the Gaussian variance of observations values (prior for how easy a transition from one state to another can happen; Gaussian distribution)	1
$W^{1/2}$	Mean of the prior on the variance of the Gaussian observations (Wishart distribution)	0.5
α	Prior on initial state probability (prior for the system starting state; Dirichlet distribution)	1
v	Variance of the prior on the variance of the Gaussian observations (Wishart distribution)	3
α_{A}	Prior on transition state probability (prior for the system transition; Dirichlet distribution)	0.5

Supplementary Table 3 | Priors Used for Order Selection by VBEM-MGHMM Analysis of the Simulated Dataset.

Parameter	Description	Value
N	Maximum order tested	4
$\mu_{I,1}$	Mean intensity state 1	172
$\mu_{I,2}$	Mean intensity state 2	172
$\mu_{I,3}$	Mean intensity state 3	172
$\mu_{I,4}$	Mean intensity state 4	172
$\mu_{bg,1}$	Mean background state 1	170
$\mu_{bg,2}$	Mean background state 2	170
$\mu_{bg,3}$	Mean background state 3	170
$\mu_{bg,4}$	Mean background state 4	170
κ	Variance of the Gaussian variance of observations values (prior for how easy a transition from one state to another can happen; Gaussian distribution)	1
$W^{1/2}$	Mean of the prior on the variance of the Gaussian observations (Wishart distribution)	0.5
α	Prior on initial state probability (prior for the system starting state; Dirichlet distribution)	1
v	Variance of the prior on the variance of the Gaussian observations (Wishart distribution)	3
α_A	Prior on transition state probability (prior for the system transition; Dirichlet distribution)	0.5

Supplementary Table 4 | TtAgo:Guide Complex Preferentially Binds to a t1G Target DNA.

	k_{on} ($M^{-1}\cdot s^{-1}$)	k_{off} (s^{-1})	molecules
Seed-matched t1A	$5.5 \pm 0.1 \times 10^7$	1.0 ± 0.01	821
Seed-matched t1T	$3.2 \pm 0.1 \times 10^7$	1.1 ± 0.01	948
Seed-matched t1C	$6.0 \pm 0.1 \times 10^7$	0.7 ± 0.01	672
Seed-matched t1G	$5.0 \pm 0.1 \times 10^7$	0.1 ± 0.01	966

Comparison of k_{on} and k_{off} of DNA-guided TtAgo with different targets. Values were derived from data collected from several hundred individual DNA target molecules (indicated as number of molecules); standard error from bootstrapping is reported.
