# Supplementary material for: An automated Bayesian pipeline for rapid analysis of single-molecule binding data
Source: Nat Commun. 2019 Jan 17;10:272. doi: 10.1038/s41467-018-08045-5 (PMC6336789; doi:10.1038/s41467-018-08045-5)
Supplement: Supplementary file 2 — Description of Additional Supplementary Files [file 41467_2018_8045_MOESM2_ESM.pdf]

Description of Additional Supplementary Files

File Name: Supplementary Movie 1
Description: Video demonstration of the pipeline's features. The analysis is performed on an example of a complete experimental dataset which consists of dark images (no illumination), images of a grid slide, images of fluorescent beads that emit in both channels, and a dataset of TtAgo binding a target DNA containing two binding sites. The demonstration shows the main modules of the pipeline: preprocessing (Electron Multiplying Charge Coupled Device (EMCCD) camera gain calibration, multichannel alignment, and drift correction), signal detection and localization (identification of target locations, detection of the binding complexes, and co-localization of the diffusible molecules at each immobilized target), and data analysis (estimation of the number of complexes bound to target molecules with multiple binding sites, calculation of association and dissociation rates, and correction for nonspecific binding of the mobile component to the glass surface). Code for the pipeline was written in MatLab. All steps are controlled via a userfriendly interface; no knowledge of MatLab syntax is required.

File Name: Supplementary Data 1
Description: DNA Oligonucleotides Used in This Study.
