# Supplementary material for: An automated Bayesian pipeline for rapid analysis of single-molecule binding data
Source: Nat Commun. 2019 Jan 17;10:272. doi: 10.1038/s41467-018-08045-5 (PMC6336789; doi:10.1038/s41467-018-08045-5)
Supplement: Supplementary file 4 — Supplementary Data 1 [file 41467_2018_8045_MOESM4_ESM.docx]

**Supplementary Data 1** | DNA Oligos Used in This Study.

| **Synthetic guide strand** | **Sequence** (Seed; **p**, 5′ monophosphate) |
| --- | --- |
| DNA guide strand,  corresponding to the first 16 nt  of let‑7a, for Alexa labeling | **p**TGA GGT AGT AGG TTG T-NH_2_ |
|  |  |
| **Substrates** | **Sequence** Bio, Biotin-6-carbon spacer; U, Alexa Fluor 647 deoxyuridine;  seed; target site/pairing to TtAgo-guide |
| Klenow polymerase template to synthesize 3′ DNA extension containing 17 Alexa Fluor 647 dyes | ATT GTT GTT ATT GTT GTT ATT GTT GTT ATT GTT GTT ATT GTT GTT ATT GTT GTT ATT GTT GTT ATT GTT GTT ATT GTT GTT ATT GTT GTT ATT GTT GTT ATT GTT GTT ATT GTT GTT ATT GTT GTT ATT GTT GTT ATT GTT GTT ATT TAC ATC TAG TTA AAC AGC GGA ACT GTG |
| Trap oligonucleotide for the preceding template (fully complementary) | CAC AGT TCC GCT GTT TAA CTA GAT GTA AAT AAC AAC AAT AAC AAC AAT AAC AAC AAT AAC AAC AAT AAC AAC AAT AAC AAC AAT AAC AAC AAT AAC AAC AAT AAC AAC AAT AAC AAC AAT AAC AAC AAT AAC AAC AAT AAC AAC AAT AAC AAC AAT AAC AAC AAT AAC AAC AAT |
| 5′-tethered, DNA target with complete complementarity to let‑7a and a 3′ DNA extension containing 17 Alexa Fluor 647 dyes | Bio-GGG TTT TAA TGA ATA CGA TTT TGT ACC AGA GTC CTT TGA TCG TGA CAA AAC AAT TGC ACT GAT AAT GAA TTG GTC TGG ATA CTA TAC AAC CTA CTA CCT CAA CCT TTT ATA CAC AGT TCC GCT GTT TAA CTA GAT GTA AAU AAC AAC AAU AAC AAC AAU AAC AAC AAU AAC AAC AAU AAC AAC AAU AAC AAC AAU AAC AAC AAU AAC AAC AAU AAC AAC AAU AAC AAC AAU AAC AAC AAU AAC AAC AAU AAC AAC AAU AAC AAC AAU AAC AAC AAU AAC AAC AAU |
| 5′-tethered, DNA target with let‑7a seed-match and a 3′ DNA extension containing 17 Alexa Fluor 647 dyes | Bio-GGG TTT TAA TGA ATA CGA TTT TGT ACC AGA GTC CTT TGA TCG TGA CAA AAC AAT TGC ACT GAT AAT GAA TTC CTC TGG ATT GAT ATG TTG GAT CTA CCT CAA CCT TTT ATA CAC AGT TCC GCT GTT TAA CTA GAT GTA AAU AAC AAC AAU AAC AAC AAU AAC AAC AAU AAC AAC AAU AAC AAC AAU AAC AAC AAU AAC AAC AAU AAC AAC AAU AAC AAC AAU AAC AAC AAU AAC AAC AAU AAC AAC AAU AAC AAC AAU AAC AAC AAU AAC AAC AAU AAC AAC AAU |
| 5′-tethered, DNA target with let‑7a seed-match plus 3′ supplementary pairing and a 3′ DNA extension containing 17 Alexa Fluor 647 dyes | Bio-GGG TTT TAA TGA ATA CGA TTT TGT ACC AGA GTC CTT TGA TCG TGA CAA AAC AAT TGC ACT GAT AAT GAA TTC CTC TGG ATT GAT AAC AAG GAT CTA CCT CAA CCT TTT ATA CAC AGT TCC GCT GTT TAA CTA GAT GTA AAU AAC AAC AAU AAC AAC AAU AAC AAC AAU AAC AAC AAU AAC AAC AAU AAC AAC AAU AAC AAC AAU AAC AAC AAU AAC AAC AAU AAC AAC AAU AAC AAC AAU AAC AAC AAU AAC AAC AAU AAC AAC AAU AAC AAC AAU AAC AAC AAU |
| 5′-tethered, DNA target with let‑7a seed-match t1A and a 3′ DNA extension containing 17 Alexa Fluor 647 dyes | Bio-GGG TTT TAA TGA ATA CGA TTT TGT ACC AGA GTC CTT TGA TCG TGA CAA AAC AAT TGC ACT GAT AAT GAA TTG GTC TGG ATT GAT ATG TTG GAT CTA CCT CAT CGT GAC AAA ACA ATT GCA CTG ATA ATG AAT TGG TCT GGA TTT GAT ATG TTG GAT AAA AAA AAA CCT TTT ATA CAC AGT TCC GCT GTT TAA CTA GAT GTA AAU AAC AAC AAU AAC AAC AAU AAC AAC AAU AAC AAC AAU AAC AAC AAU AAC AAC AAU AAC AAC AAU AAC AAC AAU AAC AAC AAU AAC AAC AAU AAC AAC AAU AAC AAC AAU AAC AAC AAU AAC AAC AAU AAC AAC AAU AAC AAC AAU |
| 5′-tethered, DNA target with let‑7a seed-match t1T and a 3′ DNA extension containing 17 Alexa Fluor 647 dyes | Bio-GGG TTT TAA TGA ATA CGA TTT TGT ACC AGA GTC CTT TGA TCG TGA CAA AAC AAT TGC ACT GAT AAT GAA TTG GTC TGG ATT GAT ATG TTG GAT CTA CCT CTT CGT GAC AAA ACA ATT GCA CTG ATA ATG AAT TGG TCT GGA TTT GAT ATG TTG GAT AAA AAA ATA CCT TTT ATA CAC AGT TCC GCT GTT TAA CTA GAT GTA AAU AAC AAC AAU AAC AAC AAU AAC AAC AAU AAC AAC AAU AAC AAC AAU AAC AAC AAU AAC AAC AAU AAC AAC AAU AAC AAC AAU AAC AAC AAU AAC AAC AAU AAC AAC AAU AAC AAC AAU AAC AAC AAU AAC AAC AAU AAC AAC AAU |
| 5′-tethered, DNA target with let‑7a seed-match t1C and a 3′ DNA extension containing 17 Alexa Fluor 647 dyes | Bio-GGG TTT TAA TGA ATA CGA TTT TGT ACC AGA GTC CTT TGA TCG TGA CAA AAC AAT TGC ACT GAT AAT GAA TTG GTC TGG ATT GAT ATG TTG GAT CTA CCT CCT CGT GAC AAA ACA ATT GCA CTG ATA ATG AAT TGG TCT GGA TTT GAT ATG TTG GAT AAA AAA ACA CCT TTT ATA CAC AGT TCC GCT GTT TAA CTA GAT GTA AAU AAC AAC AAU AAC AAC AAU AAC AAC AAU AAC AAC AAU AAC AAC AAU AAC AAC AAU AAC AAC AAU AAC AAC AAU AAC AAC AAU AAC AAC AAU AAC AAC AAU AAC AAC AAU AAC AAC AAU AAC AAC AAU AAC AAC AAU AAC AAC AAU |
| 5′-tethered, DNA target with let‑7a seed-match t1G and a 3′ DNA extension containing 17 Alexa Fluor 647 dyes | Bio-GGG TTT TAA TGA ATA CGA TTT TGT ACC AGA GTC CTT TGA TCG TGA CAA AAC AAT TGC ACT GAT AAT GAA TTG GTC TGG ATT GAT ATG TTG GAT CTA CCT CGT CGT GAC AAA ACA ATT GCA CTG ATA ATG AAT TGG TCT GGA TTT GAT ATG TTG GAT AAA AAA AGA CCT TTT ATA CAC AGT TCC GCT GTT TAA CTA GAT GTA AAU AAC AAC AAU AAC AAC AAU AAC AAC AAU AAC AAC AAU AAC AAC AAU AAC AAC AAU AAC AAC AAU AAC AAC AAU AAC AAC AAU AAC AAC AAU AAC AAC AAU AAC AAC AAU AAC AAC AAU AAC AAC AAU AAC AAC AAU AAC AAC AAU |
| 5′-tethered, DNA target with 3 complementary to let‑7a binding sites and a 3′ DNA extension containing 17 Alexa Fluor 647 dyes | Bio-GGG TTT TAA TGA ATA CGA TTT TGT ACC AGA GTC CTT TGA TCG TGA CAA AAC AAT TGC ACT GAT AAT GAA TTG GTC TAC AAC CTA CTA CCT CAG AAT TGG TCT GGA TTA CTA TAC AAC CTA CTA CCT CAG AAT TGG TCT GGA TTA CTA TAC AAC CTA CTA CCT CAA CCT TTT ATA CAC AGT TCC GCT GTT TAA CTA GAT GTA AAU AAC AAC AAU AAC AAC AAU AAC AAC AAU AAC AAC AAU AAC AAC AAU AAC AAC AAU AAC AAC AAU AAC AAC AAU AAC AAC AAU AAC AAC AAU AAC AAC AAU AAC AAC AAU AAC AAC AAU AAC AAC AAU AAC AAC AAU AAC AAC AAU |
| 5′-tethered, DNA target with 2 complementary to let‑7a binding sites and a 3′ DNA extension containing 17 Alexa Fluor 647 dyes | Bio-GGG TTT TAA TGA ATA CGA TTT TGT ACC AGA GTC CTT TGA TCG TGA CAA AAC AAT TGC ACT GAT AAT GAA TTG GTC TAC AAC CTA CTA CCT CAG AAT TGG TCT GGA TTA CTA TTG TTG GAT GAT GGA GTG AAT TGG TCT GGA TTA CTA TAC AAC CTA CTA CCT CAA CCT TTT ATA CAC AGT TCC GCT GTT TAA CTA GAT GTA AAU AAC AAC AAU AAC AAC AAU AAC AAC AAU AAC AAC AAU AAC AAC AAU AAC AAC AAU AAC AAC AAU AAC AAC AAU AAC AAC AAU AAC AAC AAU AAC AAC AAU AAC AAC AAU AAC AAC AAU AAC AAC AAU AAC AAC AAU AAC AAC AAU |
| 5′-tethered, DNA target with 1 complementary to let‑7a binding site and a 3′ DNA extension containing 17 Alexa Fluor 647 dyes | Bio-GGG TTT TAA TGA ATA CGA TTT TGT ACC AGA GTC CTT TGA TCG TGA CAA AAC AAT TGC ACT GAT AAT GAA TTG GTC TAC AAC CTA CTA CCT CAG AAT TGG TCT GGA TTA CTA TTG TTG GAT AAA AAA AGA AAT TGG TCT GGA TTA CTA TTG TTG GAT AAA AAA AGA CCT TTT ATA CAC AGT TCC GCT GTT TAA CTA GAT GTA AAU AAC AAC AAU AAC AAC AAU AAC AAC AAU AAC AAC AAU AAC AAC AAU AAC AAC AAU AAC AAC AAU AAC AAC AAU AAC AAC AAU AAC AAC AAU AAC AAC AAU AAC AAC AAU AAC AAC AAU AAC AAC AAU AAC AAC AAU AAC AAC AAU |
| 5′-tethered, DNA target with 2 let-7a seed-matched binding sites and a 3′ DNA extension containing 17 Alexa Fluor 647 dyes | Bio-GGG TTT TAA TGA ATA CGA TTT TGT ACC AGA GTC CTT TGA TCG TGA CAA AAC AAT TGC ACT GAT AAT GAA TTG GTC TGG ATT GAT ATG TTG GAT CTA CCT CAT CGT GAC AAA ACA ATT GCA CTG ATA ATG AAT TGG TCT GGA TTT GAT ATG TTG GAT CTA CCT CAA CCT TTT ATA CAC AGT TCC GCT GTT TAA CTA GAT GTA AAU AAC AAC AAU AAC AAC AAU AAC AAC AAU AAC AAC AAU AAC AAC AAU AAC AAC AAU AAC AAC AAU AAC AAC AAU AAC AAC AAU AAC AAC AAU AAC AAC AAU AAC AAC AAU AAC AAC AAU AAC AAC AAU AAC AAC AAU AAC AAC AAU |
| 5′-tethered, DNA target with 1 let-7a seed-matched binding sites and a 3′ DNA extension containing 17 Alexa Fluor 647 dyes | Bio-GGG TTT TAA TGA ATA CGA TTT TGT ACC AGA GTC CTT TGA TCG TGA CAA AAC AAT TGC ACT GAT AAT GAA TTG GTC TGG ATT GAT ATG TTG GAT CTA CCT CAT CGT GAC AAA ACA ATT GCA CTG ATA ATG AAT TGG TCT GGA TTT GAT ATG TTG GAT AAA AAA AAA CCT TTT ATA CAC AGT TCC GCT GTT TAA CTA GAT GTA AAU AAC AAC AAU AAC AAC AAU AAC AAC AAU AAC AAC AAU AAC AAC AAU AAC AAC AAU AAC AAC AAU AAC AAC AAU AAC AAC AAU AAC AAC AAU AAC AAC AAU AAC AAC AAU AAC AAC AAU AAC AAC AAU AAC AAC AAU AAC AAC AAU |
| 5′-tethered, DNA target with 2 let‑7a seed-match t1G binding sites (11 nt apart) and a 3′ DNA extension containing 17 Alexa Fluor 647 dyes | Bio-GGG TTT TAA TGA ATA CGA TTT TGT ACC AGA GTC CTT TGA TCG TGA CAA AAC AAT TGC ACT GAT AAT GAA TTG GTC TGG ATT GAT ATG TTG GAT CTA CCT CGT ATG TTG GAT CTA CCT CGA CCT TTT ATA CAC AGT TCC GCT GTT TAA CTA GAT GTA AAU AAC AAC AAU AAC AAC AAU AAC AAC AAU AAC AAC AAU AAC AAC AAU AAC AAC AAU AAC AAC AAU AAC AAC AAU AAC AAC AAU AAC AAC AAU AAC AAC AAU AAC AAC AAU AAC AAC AAU AAC AAC AAU AAC AAC AAU AAC AAC AAU |
| 5′-tethered, DNA target with 1 let‑7a seed-match t1G binding site and a 3′ DNA extension containing 17 Alexa Fluor 647 dyes | Bio-GGG TTT TAA TGA ATA CGA TTT TGT ACC AGA GTC CTT TGA TCG TGA CAA AAC AAT TGC ACT GAT AAT GAA TTG GTC TGG ATT GAT ATG TTG GAT CTA CCT CGT ATG TTG GAT GAT GGA GCA CCT TTT ATA CAC AGT TCC GCT GTT TAA CTA GAT GTA AAU AAC AAC AAU AAC AAC AAU AAC AAC AAU AAC AAC AAU AAC AAC AAU AAC AAC AAU AAC AAC AAU AAC AAC AAU AAC AAC AAU AAC AAC AAU AAC AAC AAU AAC AAC AAU AAC AAC AAU AAC AAC AAU AAC AAC AAU AAC AAC AAU |
| 5′-tethered, DNA target with 2 let‑7a seed-match t1G binding sites (56 nt apart) and a 3′ DNA extension containing 17 Alexa Fluor 647 dyes | Bio-GGG TTT TAA TGA ATA CGA TTT TGT ACC AGA GTC CTT TGA TCG TGA CAA AAC AAT TGC ACT GAT AAT GAA TTG GTC TGG ATT GAT ATG TTG GAT CTA CCT CGT CGT GAC AAA ACA ATT GCA CTG ATA ATG AAT TGG TCT GGA TTT GAT ATG TTG GAT CTA CCT CGA CCT TTT ATA CAC AGT TCC GCT GTT TAA CTA GAT GTA AAU AAC AAC AAU AAC AAC AAU AAC AAC AAU AAC AAC AAU AAC AAC AAU AAC AAC AAU AAC AAC AAU AAC AAC AAU AAC AAC AAU AAC AAC AAU AAC AAC AAU AAC AAC AAU AAC AAC AAU AAC AAC AAU AAC AAC AAU AAC AAC AAU |
